# Ureteroenteric strictures after cystectomy: Side‐specific risk factors and radiological assessment

**DOI:** 10.1002/bco2.364

**Published:** 2024-05-06

**Authors:** Simone Buchardt Brandt, Lotte Ibsen, Gitte Wrist Lam, Morten Bøttcher, Pernille Skjold Kingo, Jørgen Bjerggaard Jensen

**Affiliations:** ^1^ Department of Urology Aarhus University Hospital Aarhus Denmark; ^2^ Department of Clinical Medicine Aarhus University Aarhus Denmark; ^3^ Department of Radiology Aarhus University Hospital Aarhus Denmark; ^4^ Department of Urology Herlev and Gentofte University Hospital Copenhagen Denmark; ^5^ Department of Cardiology Regional Hospital Gødstrup Herning Denmark

**Keywords:** bladder cancer, cystectomy, ileal conduit, risk factors, side specific, ureteroenteric strictures

## Abstract

**Objective:**

To evaluate risk factors contributing to side‐specific benign ureteroenteric strictures following radical cystectomy with an ileal conduit.

**Materials and Methods:**

Data obtained from patients with bladder cancer who underwent radical cystectomy with ileal conduit surgery between 2015 and 2018 were retrospectively analysed. Imaging prior to surgery was analysed, regarding calcifications in the aorta, sarcopenia and postoperatively for length of remaining left ureter. Descriptive analyses were performed on preoperative and perioperative data, comparing patients who developed unilateral left‐ or right‐sided strictures, bilateral strictures, to those who remained free of strictures. COX regression analysis was employed to calculate crude and adjusted hazard ratio for side‐specific strictures.

**Results:**

The study included 395 patients. Strictures developed in 19% (75/395) of the patients, within a median period of 9 months: 57% (43/75) unilateral left sided, 20% (15/75) unilateral right sided and 23% (17/75) bilateral. Unilateral left‐sided stricture was associated with higher body mass index (*p* = 0.077) and hypercholesterolemia (*p* = 0.007). Right‐sided stricture was associated with a history of prior abdominal surgery (*p* = 0.029) and postoperative leakage (*p* = 0.004). Bilateral stricture was associated with smoking (*p* = 0.006) and high BMI (*p* = 0.015). The adjusted HR comparing patients with and without previous abdominal surgery was only significantly higher for right‐sided ureteroenteric strictures (HR 3.18 [95% CI: 1.11; 9.05]) compared with patients without strictures. No association was identified between strictures and preoperative aortic calcification of the abdominal aorta or sarcopenia as estimated from imaging.

**Conclusion:**

The aetiology of ureteroenteric strictures appears multifactorial. Our findings suggest that development of left‐sided stricture is influenced by factors associated with metabolic syndrome, indicating a potential role of distal ureteric ischemia. On the other hand, right‐sided stricture was more frequent in patients with previous abdominal surgery and postoperative leakage.

## 1 INTRODUCTION

The gold standard for treating localized muscle‐invasive and high‐risk non‐muscle‐invasive bladder cancer is radical cystectomy (RC) and urinary diversion.^1^ RC is associated with a high risk of both short‐ and long‐term postoperative complications.^2^ Benign ureteroenteric anastomotic stricture (UES) is a known long‐term complication after RC and is in some studies reported in up to 21% of all patients undergoing RC.^3^ The consequences of UES are numerous. Diagnosing and treating UES is not only cumbersome and an economical burden but also causes psychological strain for the patient. UES is associated with renal deterioration, upper tract infection and pain.^4^ It is important to identify risk factors to increase surveillance and awareness for patients at risk of developing UES, to ensure early diagnosis and treatment.

The pathophysiology behind UES has been suggested to include distal ischemia, which is why the left ureter, which is less resected, is more often affected by UES.^5^ However, retrospective studies have found contradicting results, regarding the length of the resected ureter and the development of UES.^6^, ^7^

UES has moreover previously been associated with different surgical techniques, comorbidities and complications. Open radical cystectomy (ORC) versus robot‐assisted radical cystectomy (RARC) has been investigated with contradicting results.^8^, ^9^, ^10^ Comorbidities have been investigated in several case series, with results indicating increased risk of UES after previous pelvis radiation, diabetes mellitus and high body mass index (BMI).^11^, ^12^, ^13^ Some surgical complications, such as leakage from the ureteroenteric anastomosis, have been associated with UES; however, the duration of stenting has not.^14^, ^15^ It is also unknown whether these factors influence the risk of UES on both the left and right ureter.

Other factors have yet to be evaluated. Calcification in the aorta could potentially be associated with UES. Therefore, the quality of the smaller vessels could be a contributing factor in the development of UES. A computerized tomography (CT) scan without contrast can be used to measure calcification of the coronary artery using the Agatston score.^16^, ^17^ In a retrospective study, atherosclerosis was evaluated using a modified Agatston score and was found to be associated with an increased risk of small bowel vascular lesions.^18^ However, investigation into a potential correlation between calcification and UES has not been done. Sarcopenia is another potential risk factor that is yet to be explored. It is a subclinical loss of skeletal muscle mass, quantified using muscle strength, evaluation of the psoas major muscle and DEXA scans.^19^, ^20^ The possible association between UES and sarcopenia has not been investigated. However, an association between sarcopenia and postoperative complications in rectal cancer resection has been found.^20^

This present study focused on assessing the risk of UES in a large cohort of patients undergoing RC with ileal conduit in a single tertiary university hospital. We aimed to determine if certain factors were associated with UES, including comorbidity, surgical techniques, complications and a radiological assessment of both the calcification of the abdominal aorta and the grade of sarcopenia. Additionally, we aimed to evaluate the risk factors when stratifying for unilateral UES (right sided and left sided) or bilateral UES.

## 2 MATERIALS AND METHODS

### 2.1 Study population

**FIGURE 1:**
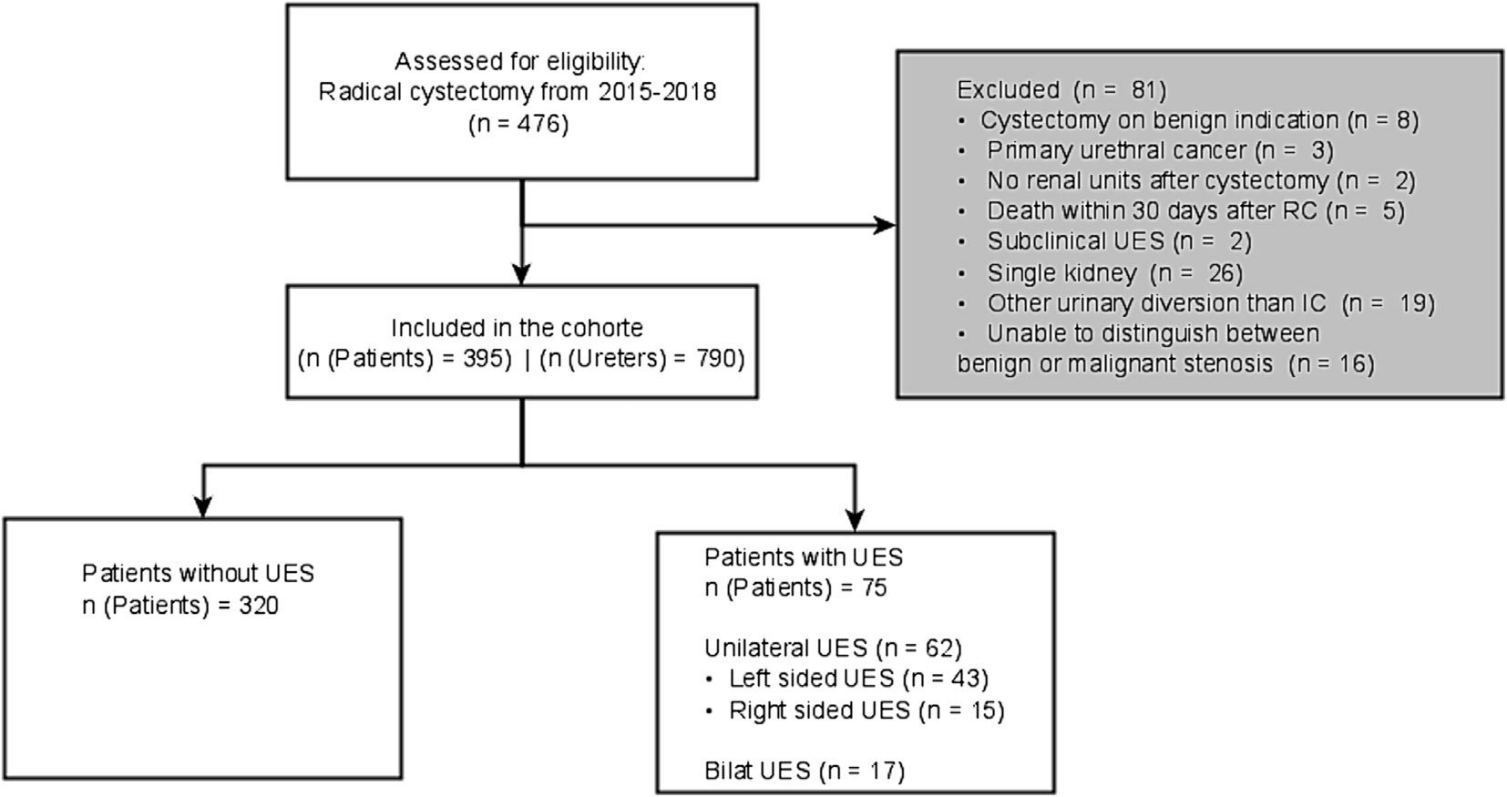
Flowchart of included and excluded patients. IC, ileal conduit; RC, radical cystectomy; UES, ureteroenteric strictures.

Data on all patients diagnosed with bladder cancer undergoing RC with an ileal conduit at Aarhus University Hospital (AUH) from January 2015 to December 2018 were included. The patient cohort is illustrated in Figure 1. Single kidney patients were excluded, to avoid bias when interpreting correlating factors with regards to the unilateral and bilateral UES subgroups. Furthermore, patients who died within 30 days after RC, and patients diagnosed with recurrence of bladder cancer in need for oncological treatment and concomitant UES were excluded, as it was difficult to distinguish between malignant and benign strictures. A total of six different highly skilled uro‐oncological surgeons subspecialized in bladder cancer performed the procedures during this period. For every patient, an extended lymph node dissection was performed according to guidelines.^1^ Approximately 15 cm of terminal ileum was used. All ureters were spatulated, stented with an 8 Fr baby‐feeding tube and anatomized using an end‐to‐side technique (ad modum Bricker) with monofilament 4‐0 absorbable running sutures. The study was approved by the Danish Data Protection Agency (1‐16‐02‐121‐20) and The Danish Patient Safety Authority (3‐3013‐2584/1).

### 2.2 Variables

Collection of data on patient demographics, comorbidity, laboratory measurements, pathological findings and imaging were conducted using electronic patient records. Data were collected using a REDCap database hosted at Aarhus University.^21^ Comorbidities included hypertension, hypercholesterolemia and diabetes, which was defined when medically treated. Postoperative leakage was registered when it was visualized on imaging and the patient underwent treatment for the leakage.

### 2.3 Outcome and follow‐up

We used the presented diagnostic criteria for UES from a previous publication.^22^ These criteria include both radiological evaluation and affected renal function either as functional decrease on renography, a creatinine increased above the threshold or symptoms reported by the patient (flank pain or upper urinary tract infections). Additionally, all UES underwent treatment with no further renal deterioration or lost in renal function. In patients who did not wish to receive any treatment of a minor UES, and no additional renal deterioration was seen. The UES was classified as subclinical and were excluded from the cohort. Competing events for UES were defined for the respective ureter, including nephrectomy, recurrence of BC and death. If none of the above‐mentioned events occurred within follow‐up, end of follow‐up was defined as last visit at the department.

### 2.4 Radiological assessment

**FIGURE 2:**
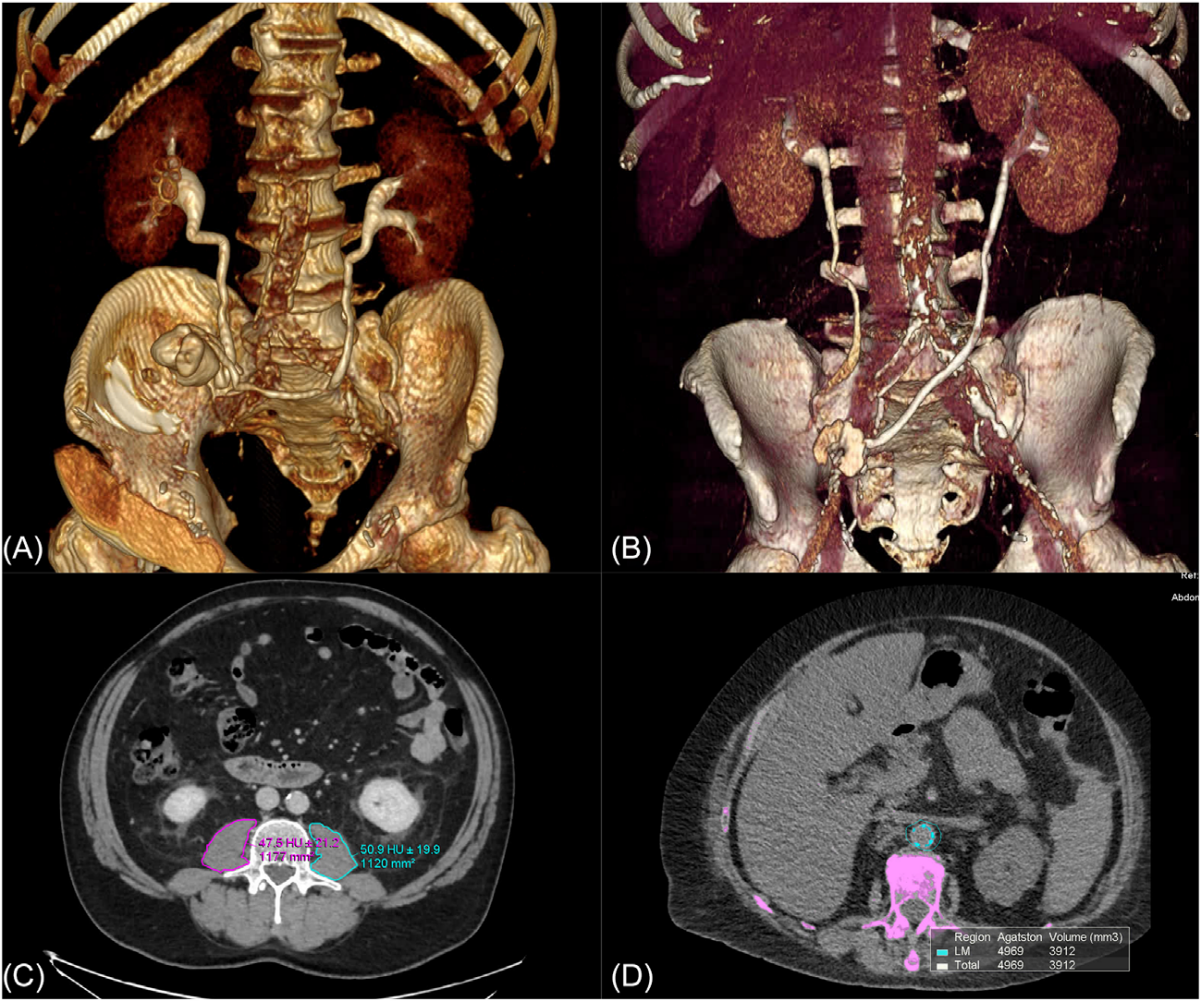
Illustration of radiological evaluation. (A) Left ureter length, total length: 174 mm. (B) Left ureter, total length: 116 mm. (C) Measurement of sarcopenia, using total psoas areas and HU of the muscle at L3 vertebra. (D) Measurement of modified Agatston score using the abdominal aorta.

Radiological evaluation was obtained using the Vitrea™ software (Canon Medical System) (Figure 2).

Length of left ureter was measured from the renal pelvis, until the ureteroenteric anastomosis (Figure 2A,B). Sarcopenia was estimated using total psoas area (mm^2^) and Hounsfield units (HU) of the muscle (Figure 2C). Measurements of the psoas major muscle areas were obtained using available preoperative CT scan images at the caudal end of L3 vertebra.^23^ Calculation of total psoas index (TPI) was performed using total psoas area and the patient's height (psoas muscle area [mm^2^]/height^2^ [m]).

Calcification of the abdominal aorta was assessed from the caudal end of the right renal artery down to aortoiliac bifurcation using available axial preoperative CT scan images (Figure 2D). Calcification of the aorta was defined as pixels with HU ≥ 130, similar to the conventional Agatston method for coronary artery calcium score.^17^

### 2.5 Statistical methods

All statistical analyses were performed using R version 4.2.2 (Boston, Massachusetts).^24^ Ureters were stratified by side of UES to determine risk factors. Rate of UES was described as a proportion with 95% confidence interval (95% CI). Continuous variables were described using medians and interquartile ranges (IQR), comparison was performed using Mann–Whitney *U* test. Categorical variables were described with numbers (*n*) and percentage, groups were compared using Fisher's exact test. Every analysis was carried out comparing the group of interest to the patients without any UES.

Postoperative complications were graded using Clavien–Dindo grade within 90 days.^25^ Comparing grades was done with and without including nephrostomy tubes.

Cox regression analysis was used to estimate crude hazard ratio (HR) with 95% CI and adjusted HR in left or right UES subgroups. Bilateral UES were excluded in this analysis. In the adjusted HR, prior variables were included in the analysis when previously proven to increase risk of UES.^13^ Three different adjusted models were carried out.

- Model 1 HR was adjusted for patient characteristics, including BMI and known comorbidities at time of RC using age‐adjusted Charlson comorbidity scale ≥3.
- Model 2 was further adjusted for previous abdominal surgery or radiotherapy in the pelvic area.
- Model 3 was further adjusted for the modality of the surgery between RARC with intracorporal urinary diversion (ICUD) and either ORC or RARC extracorporeal urinary diversion (ECUD).

An Aalen–Johansen curve was constructed to illustrate time until event of left‐sided UES adjusting for competing risk. Competing events of UES included recurrence of bladder cancer, left‐side nephrectomy and death, whereas patients were censored at last hospital visit if no event had occurred in the follow‐up period.

## 3 RESULTS

In total, 476 patients underwent RC from January 2015 to December 2018. With 81 excluded, 395 patients with bilateral ureters were included in our cohort (Figure 1). Median follow‐up was 23.2 (IQR: 10.7–28.5) months. A total of 75 patients were diagnosed with 96 UES within a median time of 9 months after RC. The rate of UES were 19% (75/395) of patients and 11.6% (92/790) of ureters after RC, with 57% (43/75) of UES unilateral in the left ureter, 20% (15/75) in the right ureter and 23% (17/75) being bilateral.

### 3.1 Side‐specific risk factors

High BMI (*p* = 0.077) and hypercholesteremia (*p* = 0.007) were more frequent in the patients with left‐sided UES, compared with patients without UES (Table 1). While unilateral right‐sided UES was associated with previous abdominal surgery (*p* = 0.029) and postoperative leakage of the anastomosis (*p* = 0.004). Side of the leakage was unspecified. The bilateral UES was associated with a high BMI (*p* = 0.015) and with smoking (*p* = 0.006) and was often males (*p* = 0.026) (Table 1).

**TABLE 1:** Characteristics of patients with and without benign ureteroenteric strictures (UES), all three UES groups are compared with patients without UES.

Characteristics	No UES (*n* = 320)	Any UES (*n* = 75)		Unilateral left‐sided UES (*n* = 43)		Unilateral right‐sided UES (*n* = 15)		Bilateral UES (*n* = 17)	
	*n*	*n*	*p* value	*n*	*p* value	*n*	*p* value	*n*	*p* value
Sex, *n* (male)	220 (69)	61 (81)	0.03	32 (77)	0.3	12 (80)	0.6	16 (94)	0.026
Age median (years)	72 (66, 77)	74 (68, 79)	0.075	75 (69, 80)	0.068	75 (68, 78)	0.5	73 (65, 77)	0.7
Smoking									
Never	63 (20)	13 (17)	0.015	5 (12)	0.12	6 (40)	0.2	2 (12)	0.006
<5 years	39 (12)	15 (20)		7 (16)		3 (20)		5 (29)	
≥5 years	120 (38)	35 (47)		21 (49)		4 (27)		10 (59)	
Smoker at time of RC	97 (30)	11 (15)		9 (21)		2 (13)		0 (0)	
BMI median (IQR)	25.6 (23.4, 28.7)	26.5 (24.8, 29.4)	0.035	26.5 (24.8, 30.3)	0.077	24.8 (23.1, 26.7)	0.5	28.1 (26.2, 29.4)	0.015
Previous pelvic radiotherapy	9 (2.8)	4 (5.3)	0.3	3 (7)	0.2	0 (0)	>0.9	1 (5.9)	0.4
Previous abdominal surgery	52 (16)	16 (21)	0.3	8 (19)	0.7	6 (40)	0.029	2 (12)	>0.9
Previously treated with BCG	26 (8.1)	7 (9.3)	0.7	4 (9.3)	0.8	2 (13)	0.4	1 (5.9)	>0.9
ASA classification									
I	31 (9.7)	6 (8.0)	0.4	4(9.3)	0.2	1 (6.7)	0.8	1 (5.9)	0.8
II	194 (61)	46 (61)		26 (60)		9 (53)		12 (71)	
III	95 (30)	22 (29)		12 (28)		6 (40)		4 (24)	
IV	0 (0)	1 (1.3)		1 (2.3)		0 (0)		0 (0)	
Age‐adjusted CCI	4 (3, 5)	4 (4, 5)	0.3	5 (4, 6)	0.036	4 (3.5, 4.5)	0.5	4 (3, 5)	0.5
Hypertension	155 (48)	39 (52)	0.6	22 (51)	0.7	9 (53)	0.7	9 (53)	0.7
Hypercholesterolemia^a^	67 (21)	24 (32)	0.041	17 (40)	0.007	2 (13)	0.7	5 (29)	0.4
DM^b^	44 (14)	11 (15)	0.8	10 (23)	0.1	0 (0)	0.2	1 (5.9)	0.7
Neoadjuvant chemotherapy	104 (33)	17 (23)	0.1	7 (16)	0.03	4 (27)	0.8	6 (35)	0.8
Preoperative hydronephrosis^c^	34 (11)	14 (19)	0.6	5 (12)	0.8	4 (27)	0.3	2 (12)	0.3
RC modality									
RARC w. ICUD	133 (42)	38 (51)	0.066	21 (49)	0.2	8 (53)	0.5	9 (53)	0.068
RARC w. ECUD	2 (0.6)	2 (2.7)		1 (2.3)		0 (0)		1 (5.9)	
ORC	185 (58)	35 (47)		21 (49)		7 (47)		7 (41)	
Stenting (days)	6 (6, 6)	6 (6, 7)	0.075	6 (6, 6)	0.7	6 (6, 6)	0.4	6 (6, 8)	0.007
pT in RC specimen									
Organ confined BC	206 (66)	62 (83)	0.003	35 (81)	0.032	14 (93)	0.023	13 (76)	0.3
Non‐organ confined BC	111 (35)	13 (17)		8 (19)		1 (6.7)		4 (24)	
Clavien–Dindo grade ≥3	107 (39)	61 (81)	<0.001	33 (77)	<0.001	12 (80)	<0.001	16 (94)	<0.001
Clavien–Dindo grade ≥3 (without nephrostomy)	89 (28)	34 (45)	0.003	18 (42)	0.058	6 (40)	0.4	10 (59)	0.011
Postoperative leakage of the ureteroenteric anastomosis^d^	13 (4.1)	11 (15)	0.002	5 (12)	0.049	4 (27)	0.004	2 (12)	0.2

Abbreviation: ASA, ASA Physical Status Classification System; BC, bladder cancer; BMI, body mass index; CCI, Charlson comorbidity index; DM, diabetes mellitus; pT, pathological T stage; RARC w. ECUD, robot‐assisted radical cystectomy with extracorporeal ileal conduit; RARC w. ICUD, robot‐assisted radical cystectomy with intracorporeal ileal conduit; RC, radical cystectomy.

^a^ Hypercholesterolemia, treated using statins.

^b^ Medically treated diabetes mellitus type 1 or 2.

^c^ Preoperative hydronephrosis is noted for relevant side (Any, left, right, bilateral).

^d^ Without knowing the side of leakage.

The adjusted HR comparing patients with and without previous abdominal surgery was greater for right‐sided UES (HR 3.18 [95% CI: 1.11; 9.05]) than left‐sided UES (HR 1.20 [95% CI: 0.55; 2.61]) when compared with patients who did not develop that side‐specific UES (Tables 2a and 2b). The majority of the previous abdominal procedures included hysterectomy, surgery for colorectal cancer and prostatectomy.

**TABLE 2:** Predictors of left‐sided (2a) and right‐sided (2b) benign ureteroenteric strictures, crude and adjusted HR with 95% CI.

Table 2a	Crude analysis			Adjusted Analysis Model 1 Adjusted for patient characteristics (BMI and Charlson comorbidity ≥3)			Adjusted Analysis Model 2 Adjusted for previous abdominal surgery or pelvic radiotherapy			Adjusted Analysis Model 3 Adjusted for surgical modality (RARC w. IC vs. RARC w. EC or ORC)		
Variables	HR	95% CI	*p* value	HR	95% CI	*p* value	HR	95% CI	*p* value	HR	95% CI	*p* value
Previous abdominal surgery	1.11	(0.51; 2.39)	0.791	1.11	(0.51; 2.40)	0.790	1.18^a^	(0.54; 2.56)	0.680	1.20^a^	(0.55; 2.61)	0.650
Previous radiotherapy	2.90	(0.89; 9.39)	0.0761	3.01	(0.93; 9.75)	0.067	3.10^b^	(0.95; 10.15)	0.062	3.33^b^	(0.99; 11.18)	0.052
Preoperative hydronephrosis	1.28	(0.50; 3.25)	0.607	1.23	(0.48; 3.12)	0.669	1.21	(0.47; 3.08)	0.695	1.28	(0.49; 3.33)	0.612
ASA classification	1.15	(0.69; 1.74)	0.595	1.03	(0.61; 1.74)	0.907	0.98	(0.59; 1.66)	0.954	1.00	(0.59; 1.68)	0.987
CCI	1.21	(1.01; 1.43)	0.0334	1.17^c^	(0.99; 1.40)	0.071	1.16^c^	(0.98; 1.37)	0.094	1.17^c^	(0.98; 1.39)	0.081
RC modality												
ORC or RARC with ECUD	ref			ref			ref			‐	‐	‐
RARC with ICUD	1.16	(0.63; 2.10)	0.637	1.11	(060; 2.04)	0.734	1.21	(0.65; 2.24)	0.552	‐	‐	‐
Stenting (days)	1.02	(0.94; 1.10)	0.651	1.02	(0.94; 1.10)	0.679	1.02	(0.94; 1.11)	0.6860	1.01	(0.93; 1.10)	0.730
Sacropenia (HUAC)	0.98	(0.93; 1.04)	0.502	0.99	(0.95; 1.04)	0.710	0.99	(0.96; 1.03)	0.744	0.99	(0.95; 1.04)	0.74
Sacropenia (TPI)	1.05	(0.88; 1.27)	0.572	1.01	(0.83; 1.23)	0.897	1.02	(0.84; 1.25)	0.816	1.02	(0.84; 1.24)	0.839
Length of remaining left ureter (mm)	1.00	(0.99; 1.01)	1	1.00	(0.99; 1.01)	0.636	0.99	(0.98; 1.01)	0.397	1.00	(0.98; 1.01)	0. 453
Modified Agatston score												
Q1	ref			ref		ref			ref			
Q2	1.32	(0.54; 3.23)	0.543	1.19	(0.48; 2.93)	0.705	1.19	(0.48; 2.95)	0.7	1.20	(0.49; 2.96)	0.694
Q3	1.52	(0.62; 3.73)	0.360	1.31	(0.53; 3.24)	0.560	1.10	(0.43; 2.83)	0.8475	1.12	(0.44; 2.89)	0.810
Q4	0.93	(0.35; 2.49)	0.892	0.84	(0.31; 2.25)	0.724	0.83	(0.31; 2.24)	0.710	0.84	(0.31; 2.27)	0.730

Table 2b	Crude analysis			Adjusted Analysis Model 1 Adjusted for patient characteristics (BMI and Charlson comorbidity ≥3)			Adjusted Analysis Model 2 Adjusted for previous abdominal surgery or pelvic radiotherapy			Adjusted Analysis Model 3 Adjusted for surgical modality (RARC w. IC vs. RARC w. EC or ORC)		
Variables	HR	95% CI	*p* value	HR	95% CI	*p* value	HR	95% CI	*p* value	HR	95% CI	*p* value
Previous abdominal surgery	3.15	(1.12; 8.84)	0.0297	3.05	(1.08; 8.67)	0.035	2.94^a^	(1.04; 8.31)	0.041	3.18^a^	(1.11; 9.05)	0.031
Previous radiotherapy	‐	‐	‐	‐	‐	‐	‐	‐	‐	‐	‐	‐
Preoperative hydronephrosis	2.26	(0.72; 7.12)	0.162	2.15	(0.67; 6.87)	0.196	2.20	(0.68; 7.13)	0.189	2.33	(0.72; 7.50)	0.157
ASA classification	1.45	(0.63; 3.38)	0.385	1.60	(0.67; 3.78)	0.287	1.69	(0.71; 3.99)	0.232	1.79	(0.75; 4.30)	0.191
CCI	0.95	(0.69; 1.32)	0.766	0.96^c^	(0.69; 1.35)	0.832	0.98^c^	(0.70; 1.36)	0.9	1.00^c^	(0.72; 1.39)	0.992
RC modality												
ORC or RARC with ECUD	ref			ref			ref			‐	‐	‐
RARC with ICUD	1.44	(0.52; 3.97)	0.48	1.48	(0.53; 4.13)	0.453	1.65	(0.59; 4.64)	0.35	‐	‐	‐
Stenting (days)	1.02	(0.88; 1.17)	0.828	1.02	(0.89; 1.17)	0.782	1.03	(0.89; 1.18)	0.72	1.02	(0.88; 1.18)	0.791
Sacropenia (HUAC)	0.99	(0.94; 1.05)	0.84	0.99	(0.90; 1.08)	0.789	0.98	(0.89; 1.09)	0.76	0.98	(0.88; 1.09)	0.692
Sacropenia (TPI)	0.84	(0.60; 1.17)	0.302	0.86	(0.61; 1.20)	0.372	0.87	(0.62; 1.22)	0.415	0.85	(0.60; 1.21)	0.368
Modified Agatston score												
Q1	ref			ref		ref			ref			
Q2	1.78	(0.45; 7.12)	0.414	1.83	(0.45; 7.52)	0.399	1.72	(0.42; 7.09)	0.452	1.74	(0.42; 7.16)	0.446
Q3	0.30	(0.03; 2.92)	0.302	0.31	(0.03; 3.10)	0.321	0.30	(0.03; 3.04)	0.311	0.31	(0.03; 3.08)	0.316
Q4	0.96	(0.19; 4.78)	0.967	0.91	(0.18; 4.78)	0.917	0.92	(0.17; 4.82)	0.918	0.94	(0.18; 4.98)	0.940

*Note*: No patients undergoing radiotherapy prior to RC developed UES; therefore, this calculation was not performed in Table 2b.

Abbreviations: CCI, Charlson comorbidity index (age adjusted); HUAC, Hounsfield unit average calculation; ORC, open radical cystectomy; RARC w. ECUD, robot‐assisted radical cystectomy with extracorporal ileal conduit; RARC w. ICUD, robot‐assisted radical cystectomy with intracorporal ileal conduit; TPI, total psoas index.

^a^ Not adjusted for previous abdominal surgery.

^b^ Not adjusted for previous pelvis radiotherapy.

^c^ Not adjusted for CCI > 3.

### 3.2 Length of left ureter

Length of the left ureter postoperatively was measured in 91% (330/363) of patients without UES and with left‐sided UES. Median length of the ureter for patients without UES were 137 (IQR: 120–157) mm, compared with patients with left‐sided UES 135 (IQR: 116–156) mm, not significantly different (*p* = 0.8).

### 3.3 Calcification

The modified Agatston score of the abdominal aorta showed a median of 3321 (IQR: 1116.0–6507.0). Imaging to measure calcification was available in 87.3% (345/395) of the total cohort. Patients with UES had a median of 2989 (IQR: 2129–5558), patients who did not develop UES had a median of 3537 (IQR: 1033–6805), not significantly different (*p* ≥ 0.9). Tables 2a and 2b show the HR in the left‐ and right‐sided UES subgroups.

### 3.4 Sarcopenia

The psoas major muscle was estimated to have a median TPI of 5.3 (IQR: 4.4–6.3) cm^2^/m^2^ with a Hounsfield unit average calculation (HUAC) of 23.5 (IQR: 20–26.2). Comparing UES patients with patients without UES shows non‐significant differences in size and density of the muscle. Imaging to measure sarcopenia was available in 99% (391/395) of the total cohort.

**FIGURE 3:**
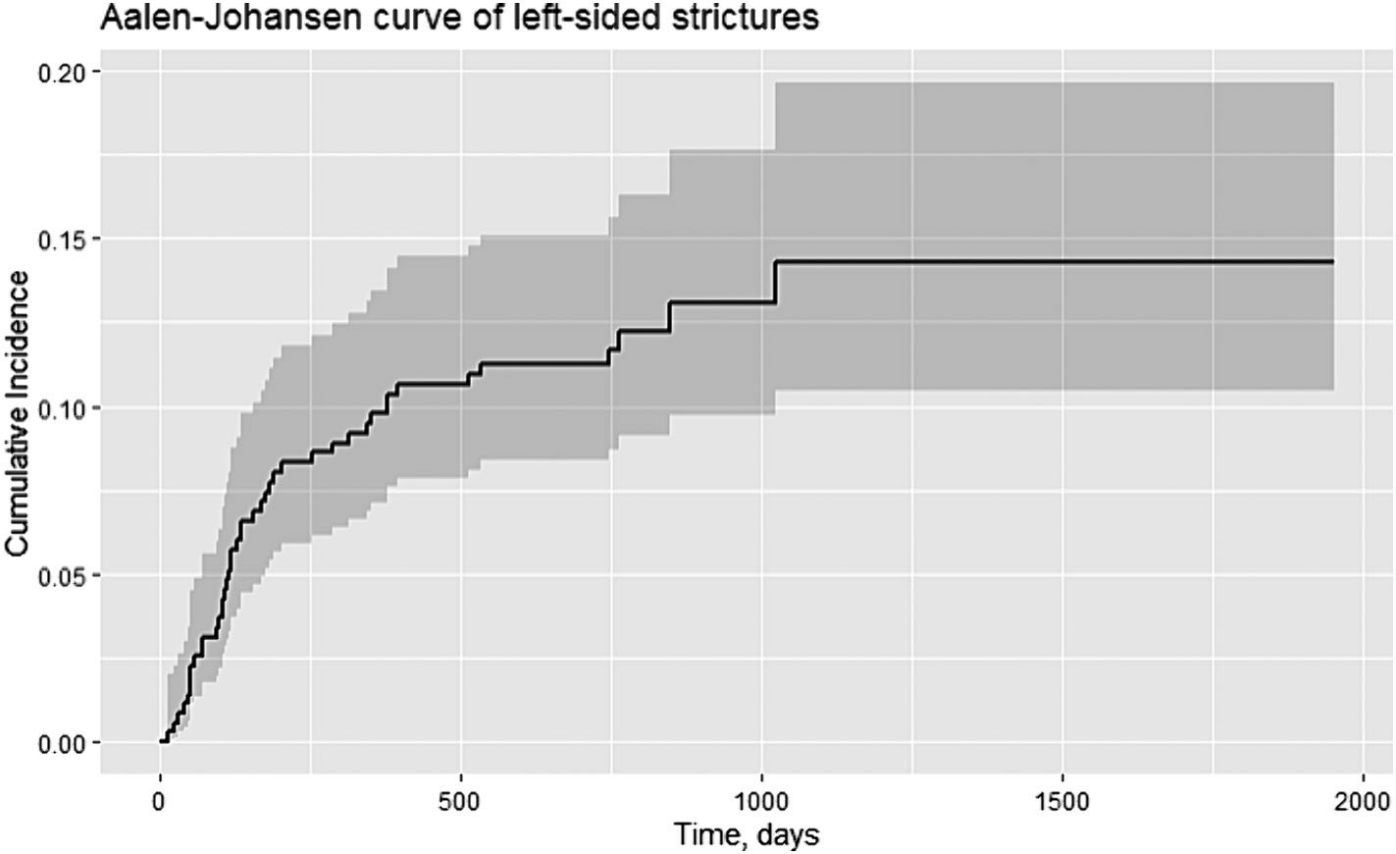
Aalen–Johansen curve of unilateral left‐sided stricture.

Time until event is illustrated in Figure 3, using cumulative incidence function adjusting for competing risk.

## 4 DISCUSSION

In this homogeneous cohort of bladder cancer patients treated with RC and IC, we found that 19% of patients were diagnosed with one or more UES, the majority within the first year and in the left ureter. In the Aalen–Johansen curve in Figure 3, it is illustrated that the risk of UES is low 2 years postoperatively.

Several factors in the present study were associated with unilateral left‐sided UES. This included high BMI, and some comorbidities, specifically hypercholesterolemia, increased the rate of left‐sided UES following RC. These factors could indicate a component of metabolic syndrome, with the related vascular consequences.^26^ The association between postoperative complications and metabolic syndrome has previously been investigated in a meta‐analysis; they found increased mortality, infections, cardiac events and readmissions following surgery.^27^ However, it is unknown if patients in the current cohort fulfilled the three out of five of criteria for metabolic syndrome, including elevated blood pressure, elevated triglycerides, low high‐density lipoprotein, raised fasting glucose and central obesity.^28^

In contrast, unilateral right‐sided UES patients (15/395), although less frequent, had significantly more cases of previous abdominal surgery and leakage of the ureteroenteric anastomosis. Association between abdominal surgery and UES has previously been proven, however not exclusively with right‐sided UES.^13^ Additionally, right‐sided hydronephrosis at time of RC was more frequent, in the right‐sided UES group. In summary, these factors indicate that patients rarely develop right‐sided UES unless it is associated with challenging surgery.

In the present study, all three UES subgroups were significantly associated with leakage of the ureteroenteric anastomosis, although the side of leakage was unknown. This association has been confirmed in previous studies and presumably indicates that the leakage starts an inflammatory reaction in the tissue.^14^ Postoperative complications were associated with all subgroups of UES. However, it is important to note that this result may be biased due to the registration of nephrostomy tubes as Clavien–Dindo grade IIIa. Still, when analysing the postoperative grading without considering nephrostomy tubes, the association with UES persisted.

The risk of UES after ORC compared with RARC has been discussed; however, even though the randomized RAZOR trial was not powered to estimate the risk of UES, they found no differences in rates of UES between the ORC and RARC with ECUD.^10^ Retrospective studies have however found contradicting results.^8^, ^9^ In the present study, the crude HR of UES between RARC ICUD and ORC or RARC ECUD was 1.16 (0.63; 2.10) for left UES and 1.44 (0.52; 3.97) for right‐sided UES, indicating higher risk of UES when the RC includes ICUD but not significantly. RARC has previously been associated with an increased risk of UES compared with ORC.^29^ However, the reason for this is unknown. It could be explained by a lack of haptic feedback or increasing numbers of sutures, due to the increased visualization of the anastomosis reducing the vascularization in the area. Inflammatory infiltration has been found significantly more in the left ureter compared with the right in a porcine study.^30^ They also found the grade of inflammatory infiltration was higher when RC was performed as RARC compared with ORC. In the present study, the robot‐assisted operation was introduced in 2012; thus, the initial learning curve after introducing the robot cannot explain this tendency, as argued by others.^8^

A modified Agatston score adapted from the conventional have been used in numerous ways. A previous study found correlation between arteriosclerosis and small bowel lesions, measured from the level of the diaphragm to the aortoiliac bifurcation.^18^ In the present study, we found no association between the modified Agatston score and UES. We measured from the right renal artery to the aortoiliac bifurcation; this makes it difficult to compare results with those of others.

Radiological evaluation of the length of the remaining left ureter has not previously been performed. This present study found no association between length of the remaining left ureter and the risk of developing left‐sided UES. Comparing length of ureter between patients might not be relevant with a large variation. However, in Adjusted Models 2 and 3, the HR is adjusted for BMI and shows no effect on the HR. It could also indicate that this measurement in postoperative CT scans is unprecise or that the remaining length of left ureter is not relevant for the development of UES compared with the vascularization itself. Others have found that length of resection influence the risk of UES.^7^

Sarcopenia has been associated with postoperative complications in previous studies.^20^, ^23^ Sarcopenia is a subclinical loss of skeletal muscle mass and is associated with increased morbidity and mortality after surgery.^19^, ^20^ This study is the first to evaluate sarcopenia in relation to the development of UES and used TPI and HUAC prior to RC. However, sarcopenia was not found to be a predictor of UES.

### 4.1 Study limitations

The present study is limited by the retrospective design and possibly missing data. This includes information on the side of leakage of the anastomosis, CT scans on all patients and specific blood tests, to investigate undiagnosed dyslipidaemia and diabetes. Measurement of length of remaining left ureter, sarcopenia and aortic calcification using the modified Agatston score are limited to a single investigator. It would have strengthened the study, if more investigators had evaluated the scans followed by an inter‐observer agreement analysis. Additionally, the low number of especially right and bilateral UES limits the power of our estimates.

Despite the limitations of the present study, this is the largest cohort study of RC with ileal conduit to investigate UES grouped on side‐specific development of UES.

## 5 CONCLUSION

In this study in patients after RC, we found a rate of UES of 19% following RC with ileal conduit, with the majority on the left side. UES appears to have a multifactorial aetiology, with a low risk after the initial 2 years post‐surgery. This study suggests that factors associated with metabolic syndrome, such as high BMI and hypercholesteremia, were linked to unilateral left‐sided UES. Unilateral right‐sided UES, on the other hand, was associated with challenging surgery, as a high prevalence of prior abdominal surgery and anastomotic leakage were documented in this subgroup. Bilateral UES was associated with high BMI and a history of smoking. No association was identified between UES and preoperative aortic calcification or psoriatic sarcopenia.

The findings indicates that the aetiology of UES varies depending on side, and further studies with side‐specific evaluation are needed for a definitive understanding of the development of UES.

## AUTHOR CONTRIBUTIONS

**Simone Buchardt Brandt:** Conceptualization; methodology; formal analysis; investigation; writing—original draft; visualization. **Lotte Ibsen:** Methodology; writing—review and editing; supervision. **Gitte Wrist Lam:** Writing—review and editing; supervision. **Morten Bøttcher:** Methodology; resources; writing—review and editing. **Pernille Skjold Kingo:** Conceptualization; writing—original draft; supervision. **Jørgen Bjerggaard Jensen:** Conceptualization; methodology; resources; writing—original draft; supervision.

## CONFLICT OF INTEREST STATEMENT

None.
